# The 3D nuclear conformation of the major histocompatibility complex changes upon cell activation both in porcine and human macrophages

**DOI:** 10.1186/s12860-021-00384-4

**Published:** 2021-09-14

**Authors:** Florence Mompart, Alain Kamgoué, Yvette Lahbib-Mansais, David Robelin, Agnès Bonnet, Claire Rogel-Gaillard, Silvia Kocanova, Martine Yerle-Bouissou

**Affiliations:** 1 GenPhySE, Université de Toulouse, INRAE, ENVT, 1388 GenPhySE, 24 Chemin de Borde Rouge, 31326 Cedex Castanet-Tolosan, France; 2 Laboratoire de Biologie Moléculaire Eucaryote (LBME), Centre de Biologie Intégrative (CBI), CNRS, UPS, University of Toulouse, 31062 Toulouse, France; 3 Université Paris-Saclay, INRAE, AgroParisTech, GABI, 78350 Jouy-en-Josas, France

**Keywords:** Nuclear architecture, Gene expression, Major histocompatibility complex, Macrophage, LPS/IFNγ activation, 3D-FISH

## Abstract

**Background:**

The crucial role of the major histocompatibility complex (MHC) for the immune response to infectious diseases is well-known, but no information is available on the 3D nuclear organization of this gene-dense region in immune cells, whereas nuclear architecture is known to play an essential role on genome function regulation. We analyzed the spatial arrangement of the three MHC regions (class I, III and II) in macrophages using 3D-FISH. Since this complex presents major differences in humans and pigs with, notably, the presence of the centromere between class III and class II regions in pigs, the analysis was implemented in both species to determine the impact of this organization on the 3D conformation of the MHC. The expression level of the three genes selected to represent each MHC region was assessed by quantitative real-time PCR. Resting and lipopolysaccharide (LPS)-activated states were investigated to ascertain whether a response to a pathogen modifies their expression level and their 3D organization.

**Results:**

While the three MHC regions occupy an intermediate radial position in porcine macrophages, the class I region was clearly more peripheral in humans. The BAC center-to-center distances allowed us to propose a 3D nuclear organization of the MHC in each species. LPS/IFNγ activation induces a significant decompaction of the chromatin between class I and class III regions in pigs and between class I and class II regions in humans. We detected a strong overexpression of TNFα (class III region) in both species. Moreover, a single nucleus analysis revealed that the two alleles can have either the same or a different compaction pattern. In addition, macrophage activation leads to an increase in alleles that present a decompacted pattern in humans and pigs.

**Conclusions:**

The data presented demonstrate that: (i) the MHC harbors a different 3D organization in humans and pigs; (ii) LPS/IFNγ activation induces chromatin decompaction, but it is not the same area affected in the two species. These findings were supported by the application of an original computation method based on the geometrical distribution of the three target genes. Finally, the position of the centromere inside the swine MHC could influence chromatin reorganization during the activation process.

**Supplementary Information:**

The online version contains supplementary material available at 10.1186/s12860-021-00384-4.

## Background

The immune system plays a central role in mammals, not only in health maintenance but also in pathogenesis. There are several ways of protecting a human or an animal against invasion by pathogens. The first line of defense is provided by an innate immune response consisting of barriers such as skin, tears, saliva and mucus, as well as an inflammatory response. This is closely followed by defensive mechanisms due to adaptive immune responses that include both a humoral response produced by antibodies, and a cell-mediated response produced by T cells that have the ability to destroy other cells. The immune system also includes other key anti-infectious actors that are able to destroy infectious agents, known as phagocytic cells, such as monocyte-derived macrophages [1] and neutrophils [2]. Neutrophils are the first cells to intervene at infection sites but have a short lifespan and are taken over by monocytes that differentiate into macrophages and phagocyte both pathogens and apoptotic neutrophils. To do this, these immune cells undergo a series of distinct functional changes both at the cytoplasm and nucleus levels. Most of these changes imply variations in gene expression revealed by transcriptomic analyses [3].

The cell-mediated adaptive immune response is regulated by the major histocompatibility complex (MHC). The MHC it is one of the most gene-dense regions in mammals and has been divided into three regions referred to as class I, II and III [4]. The class I and class II regions include histocompatibility genes that encode proteins involved in the adaptive immune response, through their respective presentations of endogenous and exogenous peptide antigens, to circulating T lymphocytes [5]. The class III region comprises many important immune-defense genes such as the tumor necrosis factor gene families and components of the complement cascade. In humans, this complex (HLA Human Leucocyte Antigen) resides in the short arm of chromosome 6 and spans over 3.6 Mb [6]. In pigs, the MHC complex, or swine leukocyte antigen (SLA), is located on chromosome 7 (SSC7) [7] and spans between 2.4 and 2.66 Mb, depending on the haplotype [8]. The class I and class III regions are located on SSC7p1.1, while the class II region is located on SSC7q1.1 [9]. This physical assignment of the swine MHC spanning the centromere of SSC7 is unique among mammals studied to date. The SLA class I, class III and class II regions were found to span approximately 1.06 Mb, 0.67 Mb and 0.46 Mb, respectively (Scrofa11.1 assembly), making the swine MHC the smallest among mammalian MHC examined so far. Over 150 loci have been identified in the entire SLA region, and at least 121 genes are predicted to be functional [10] and are reviewed in [8]. The SLA reference sequence comprises the entire class I, class II, and class III regions, and a BAC contig that covers the entire region with the exception of the centromere has been built [10, 11].

Numerous studies have shown that chromatin is organized into hierarchical 3D structures that are thought to play a role in gene regulation [12–14]. Insights into the functions and 3D structures of genomes have been mainly derived from microscopy techniques such as 3D fluorescence in situ hybridization (3D FISH), molecular approaches including chromosome conformation capture technologies (3C and its derivatives) and, more recently, ligation-free methods (reviewed in [15]). As of this time, the spatial distribution of the genome is explored at the genome-wide scale by high-throughput chromosome conformation capture (Hi-C) that provides genome-wide maps of contact frequencies [16]. However, these methods performed on populations of millions of cells give an average view of what is happening in the cell population. Single-cell Hi-C experiments and super-resolution imaging [17–20] and, more recently, Hi-C combined with high-throughput fluorescence in situ hybridization (hiFISH) [21], have revealed the high degree of cell-to-cell and allele-to-allele heterogeneity in spatial genome organization, emphasizing the benefits of combining single-cell and cell population approaches.

The position of single genes within the nucleus has been widely investigated. While their non-random positioning has been largely demonstrated, the link with their functional status is still being debated [22, 23]. Similarly, a link between relative gene position within a chromosome territory (interior: less active or repressed; exterior or looped out: more active), and transcription has been established in numerous studies [24–27]. In this context, we previously investigated the spatial arrangements of genes differentially expressed (up- and down-regulated) in porcine macrophages when the immune cells are activated by LPS (lipopolysaccharide)/IFNγ [28]. Our results demonstrated that there is not a systematic relocalization of these genes in the nuclear space. Three of the four up-regulated genes relocalized in the nucleus and/or relative to their chromosome territories (CT), while the four down-regulated genes did not change their positions. We found the same results in neutrophils, another type of immune cell, but not in fibroblasts, except for TNFα, which retains its tendency to be at the edge or outside of its CT in the three cell types tested. These results suggest that relocalization of genes differentially expressed in response to LPS/IFNγ activation is gene- and cell type-specific, as well as being closely linked to the entire up-regulation status of their chromosomal regions [28]. TNFα is located in a region of prime importance for the immune response since it contains the MHC. However, no information is available on the 3D organization of this particular and important region.

In the present study, we focused our analysis on this region to investigate its 3D conformation in macrophages. In terms of physical organization, the human and porcine MHCs harbor an important difference due to the presence of the centromere inside the complex in swine where it separates the class II from the class III region. The analysis was done on both porcine and human macrophages to determine if this particular organization in swine has an impact on the 3D conformation of the MHC and on its plasticity. We also investigated whether this conformation changes when macrophages are activated by LPS/IFNγ. To address these questions, we selected Bacterial Artificial Chromosomes (BACs) containing genes that map to each of the three MHC regions in humans and pigs in order to examine their spatial arrangement. This was performed by 3D-FISH on structurally preserved macrophages in combination with confocal microscopy and 3D image analyses. The expression of the three genes selected to represent each MHC region was assessed by quantitative real-time PCR in resting and activated macrophages to ascertain whether a response to a pathogen aggression modifies the expression level of the selected genes and the 3D organization of the complex. Finally, we assessed 3D folding of the MHC by application of a mathematical approach that allowed us to confirm our observations.

## Results

### Selection of BACs containing MHC genes

**Fig. 1:**
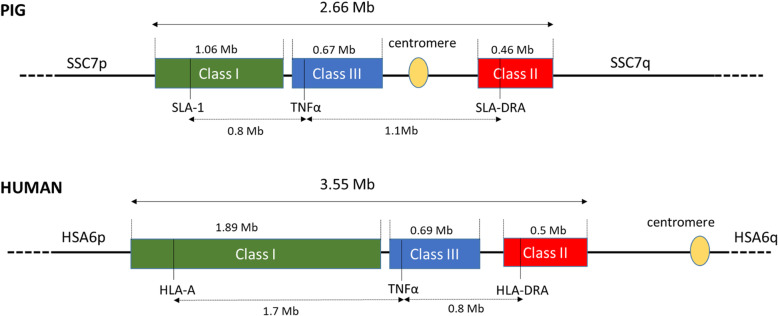
Comparative genomic organization of the swine and the human major histocompatibility complex (MHC). The genes that were used in this study to represent each MHC region are indicated with their relative position in their corresponding class. The swine leucocyte antigen (SLA) class I and III regions reside on the short arm of chromosome 7 (SSC 7: *Sus scrofa* 7) and are separated from the class II region located on the long arm by the centromere. The genomic distances are those reported in [8] and are taken from the swine genome assembly 11.1. The human leucocyte antigen (HLA) complex resides on the short arm of chromosome 6. The genomic distances are taken from the human genome assembly GRCh38.p13

In order to investigate the 3D nuclear organization of the MHC in macrophages and to analyze the effects of LPS/IFNγ activation on both, the nuclear organization and the expression of target genes, we selected BACs containing genes specific to each MHC region in pigs and in humans. Comparative analysis of the MHC in mammalian species has revealed some species-specific features, the most notable being the size of the class I region, which is almost two times bigger in humans (1.89 Mb) compared to pigs (1.06 Mb) (Fig. 1), and the position of the centromere that interrupts the SLA complex between class III and II regions, whereas it is outside in the HLA complex. However, there are also large regions of shared syntenies and sequence conservation between the SLA and the HLA complex [10] that have facilitated the choice of the homologous genes representative of each MHC class. We selected SLA-1 and HLA-A based on their genomic positions and functionality as classical class I genes, TNF**α,** the master regulator of inflammatory cytokine production in both species for the MHC class III region and, finally, SLA-DRA and HLA-DRA involved in peptide presentation to CD4 T cells for the MHC class II region (Fig. 1). The presence of the genes in the BAC clones selected for the FISH experiments was verified by PCR, and the chromosomal locations of all BACs were controlled by 2D-FISH on metaphase spreads, both in porcine and in human cells. As expected, the BACs containing SLA-1 (class I region) and TNF**α** (class III region) were found on the short arm of porcine chromosome 7 (SSC7p), and the BAC containing SLA-DRA (class II region) mapped to the long arm (SSC7q), while the three BACs containing the human genes were found on the short arm of human chromosome 6 (HSA6p) (Table 1; Additional file 1: Fig. S1).

**Table 1:** Primers for BAC clones verification by PCR and for qPCR experiments

Target genes (MHC Class)	Localization	Primer sequences	Genomic DNA amplicon size (bp)^a^	cDNA Amplicon size (bp) (Accession numbers)
SLA-1 (I)	SSC 7p1.1	TGGTGGCTGGAGTTGTGATC	515	66 (NM_001097431)
		CCTGAGTGTAGCTCCCTCCT		
SLA-DRA (II)	SSC 7q1.1	CCCTCAACCGAGGATGTCTA	432	126 (NM_001113706)
		AGCACACACGGTGTTCTCTG		
TNFα (III)	SSC 7p1.1	ACTGCACTTCGAGGTTATCGG	887	118 (NM_214022)
		GGCGACGGGCTTATCTGA		
B2M	SSC 1q1.7	TCATCCAACCCAGATGCA	1733	162 (NM_213978)
		TTCTACCTTCTGGTCCACACTGA		
HLA-A (I)	HSA 6p22.1	TACAACCAGAGCGAGGC	373	132 (NM_002116)
		CTCGTTCAGGGCGATGTAAT		
HLA-DRA (II)	HSA 6p21.32	CCCTCAACTGAGGACGTTTA	393	105 (NM_019111)
		AGTCTCTGGGAGAGGGCTTG		
TNFα (III)	HSA 6p21.33	GCTGCACTTTGGAGTGATCG	916	123 (NM_000594)
		TGGGCTACAGGCTTGTCACT		
B2M	HSA 15q21.1	TACACTGAATTCACCCCCACTG	2020	143 (NM_004048.2)
		TCCAATCCAAATGCGGCATC		

^a^Genome reference sequences: swine Sscrofa11.1; human GRCh38p13

### Radial positioning of class-specific MHC genes

**Fig. 2:**
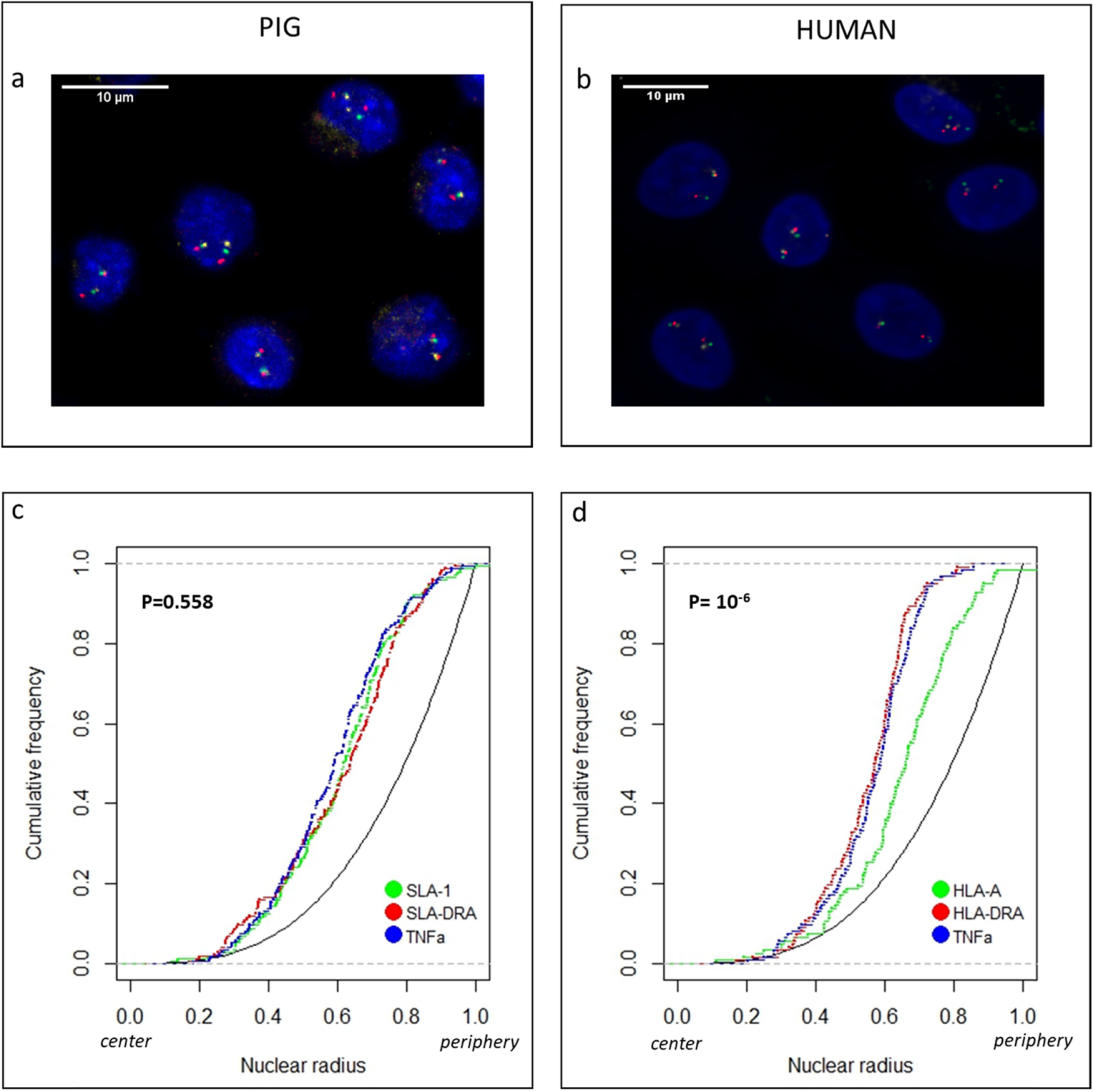
**a-b** Maximum intensity projections of confocal stacks of 3D-FISH experiments carried out with the three BAC probes representative of each MHC region co-hybridized on structurally preserved porcine (**a**) and human (**b**) macrophage nuclei: BAC containing class I is labeled in green with Alexa 488; BAC containing class II is labeled in red with Alexa 568; and BAC containing class III is labeled in yellow with Alexa 633. DNA was counterstained with DAPI. Bar scale: 10 μm. **c-d** Radial distribution of MHC genes in resting porcine (**c**) and human (**d**) macrophages. Cumulative frequency graphs of the radial position for each MHC class: SLA-1 and HLA-A for class I in green; SLA-DRA and HLA-DRA for class II in red; and TNFα for class III in blue. The cumulative frequency curve of a random distribution (P(X < d) = d3 for 0 < d < 1 where X is the random radial position) is shown in black in each graph for comparison. Pairwise comparisons (*p*-values) of cumulative radial distribution (Student’s t-test) are indicated in each graph. The center of the nucleus corresponds to 0 on the X-axis and the periphery to 1

To first determine the localization of the MHC in the macrophage nuclei, we hybridized the BACs specific to the three MHC regions in 3D-FISH experiments on structurally preserved porcine and human macrophages. For the purpose of simplification, the BAC probes will be designated by the gene chosen to represent each MHC region. Figure 2 provides examples of typical patterns observed in porcine (Fig. 2a) and human (Fig. 2b) macrophage nuclei after hybridization with the MHC probes. After each 3D experiment, we acquired serial optical sections using confocal microscopy from around 60 to 88 nuclei (see Methods). Images were processed with NEMO software [29]. Distances between the probe signal centers and the nucleus center were measured for the three selected genes and were individually normalized to the nucleus radius of each cell to enable comparisons between different nuclei*.* Cumulative frequency graphs of the radial positions for each gene in porcine and human macrophages demonstrated their non-random radial nuclear organization (Fig. 2c-d)*.* No difference in radial positioning was observed between the three genes in porcine marcophages (Fig. 2c). We observed that the genes tend to occupy an intermediate position. The results are quite different in human macrophages since HLA-DRA (class II) and TNFα (class III) both occupy an intermediate position, whereas HLA-A (class I) shows a significantly more peripheral position (Fig. 2d). These results suggest that the HLA and SLA complexes probably have a different 3D organization.

### Gene-to-gene distances in the MHC and spatial organization

**Fig. 3:**
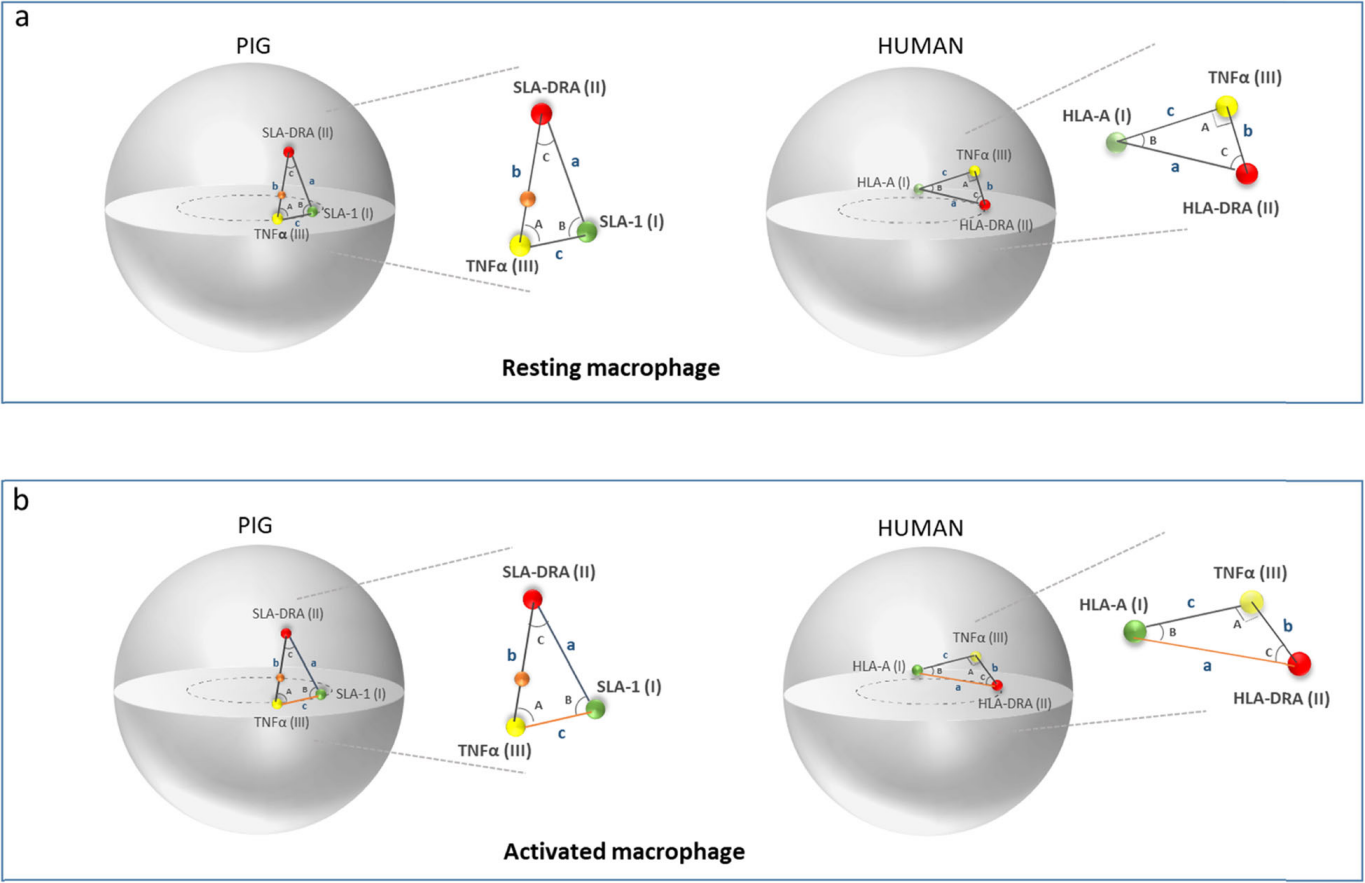
Schematic representation of the 3D organization of the MHC in resting (**a**) and LPS/IFNγ activated (**b**) porcine and human macrophages based on the 3D interprobe distance measurements. a: distance class I-class II; b: distance class II-class III; c: distance class I-class III; A: angle class III; B: angle class I; C: angle class II; chromosome 7 centromere: orange point. The 3D distances that increase with macrophage activation are indicated by a red line

In order to study the 3D organization of the MHC in both species, we measured the 3D interprobe distances: (i) SLA-1/HLA-A (class I)-SLA-DRA/HLA-DRA (Class II) distance, denoted a; (ii) SLA-DRA/HLA-DRA (class II)-TNFα (class III), denoted b*;* and (iii) SLA-1/HLA-A (class I)–TNFα (class III), denoted c (Fig. 3a). We normalized each measured distance to the nucleus diameter of each cell to allow comparisons between cells. In addition, using these 3D measurements, we calculated all the internal angles (Fig. 3a; Additional file 2: Table S1). We observed that in resting porcine macrophages, the distances a and b are equal, and the distance c is almost twice as small. Similarly, angles A and B are equal, and angle C is three times smaller. In humans, the situation is quite different. It is indeed the b distances that are about two times smaller than the a and c distances. Concerning the angles, A and B are not equal and angle B is the smallest of the three angles. These results add an additional argument for a different 3D organization of the MHC in resting pig and human macrophages (Fig. 3a).

**Fig. 4:**
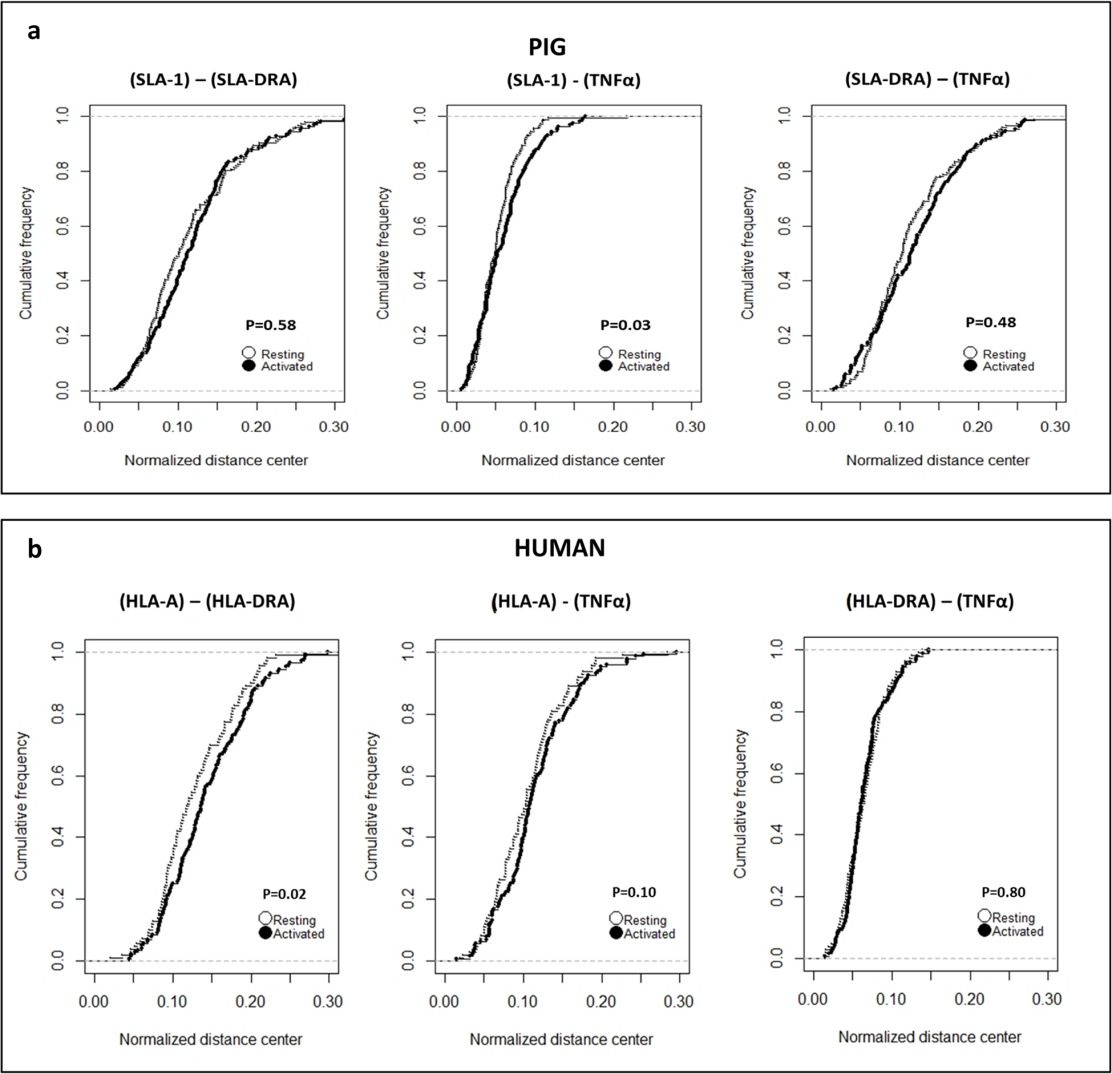
**a-b** Analysis of LPS/IFNγ activation effects on MHC gene-to-gene 3D distances in porcine and human macrophages. Cumulative frequency graphs of the normalized center-to-center distances between MHC: (i) class I-class II [(SLA-1)-(SLA-DRA); (HLA-A)-(HLA-DRA); (ii) class II-class III [(SLA-DRA)-TNFα; (HLA-DRA)-TNFα]; and (iii) class II-class III [(SLA-1)-TNFα; (HLA-A)-TNFα]. Pairwise comparisons (p-values) of cumulative 3D distance distributions in resting and activated macrophages (Student’s t-test) are indicated in each graph

We next investigated whether a response to a pathogen aggression modifies the 3D organization of the MHC. To answer this question, we carried out the same 3D measurements between the MHC-specific genes in macrophages that have been activated by LPS/IFNγ [28]. We compared these measurements in both states (resting and activated). This analysis revealed that the SLA-1/TNFα distance (c) significantly increases (*p* = 0.03) when the porcine macrophages are activated, suggesting a reorganization of the chromatin in this region (Figs. 3b and 4a). The distances between SLA-1/SLA-DRA and between TNFα/SLA-DRA do not change (Fig. 4a). Considering the unique feature in the organization of the MHC due to the presence of the centromere separating the class II and class III regions, we completed the analysis by measuring the 3D center-to-center distances between TNFα and the centromere and between the centromere and SLA-DRA in resting and activated porcine macrophages. The comparison of these measurements in the two states reveals no difference (Additional file 3: Fig. S2). In human macrophages, only one distance significantly increases when the macrophages are activated, but this is the HLA-A/HLA-DRA distance (a) (*p* = 0.02) (Figs. 3b and 4b), reinforcing the hypothesis of a different organization of the complex and chromatin conformation changes due to LPS/IFNγ activation in humans and pigs.

### Gene expression analysis for the MHC-specific genes

**Fig. 5:**
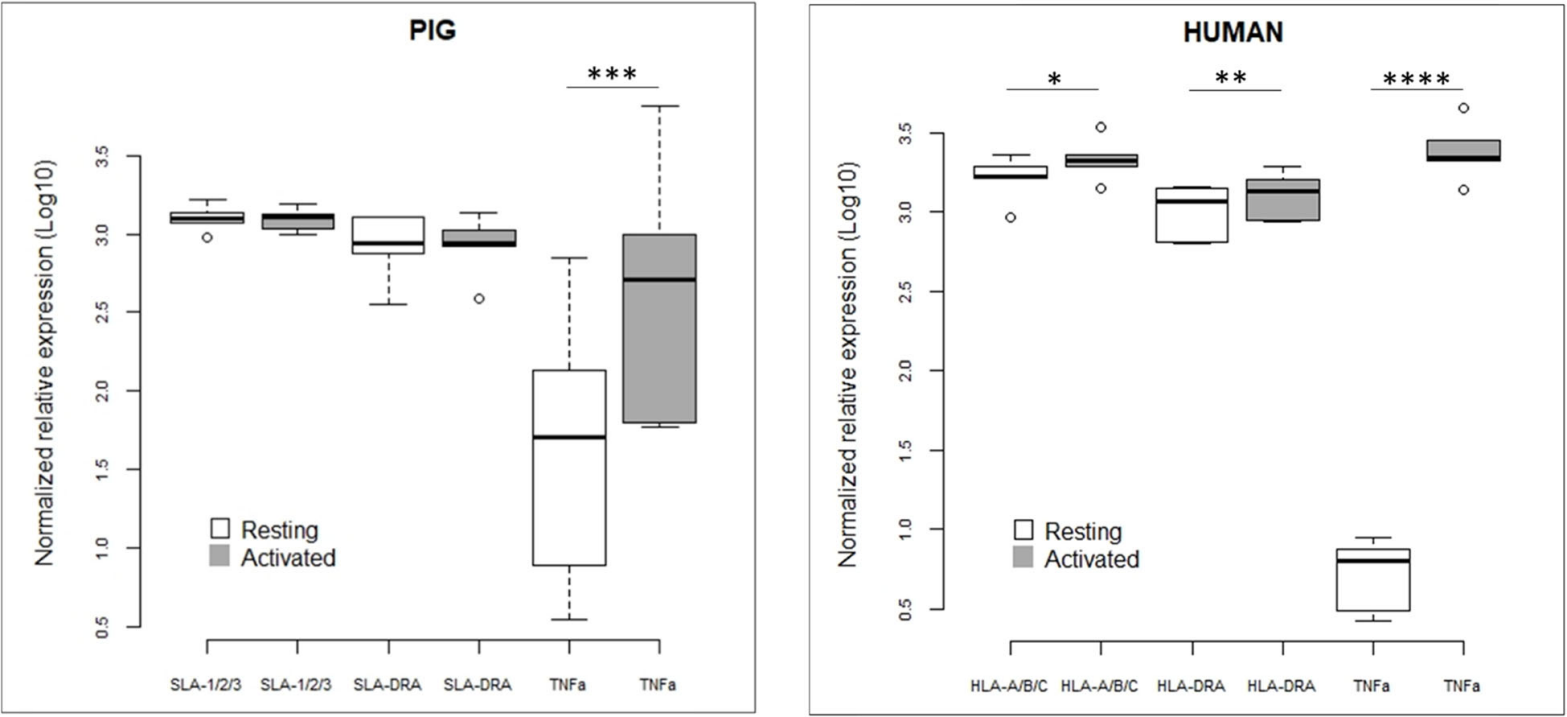
Normalized relative expression graphs in porcine and human macrophages. mRNA expression level, analyzed by RT-qPCR both in resting and activated macrophages, were pairwise compared with one-way ANOVA models with repetitions. *P*-value class: *p < 0.05; **p < 0.01; ***p < 0.001, ****p < 10^− 4^

In order to assess whether the modifications of the 3D organization during the activation process are associated with a change in gene expression, we extracted total RNA from resting and activated macrophages for both species. We quantified gene expression level by real-time PCR for the genes selected in the MHC. These are SLA-1 and HLA-A for MHC class I, SLA-DRA and HLA-DRA for class II, and TNFα for class III, both in pigs and humans. For MHC classical class I genes, due to the very high sequence homology between SLA-1, SLA-2 and SLA-3 in pigs and, similarly, for their functional orthologs HLA-A, HLA-B and HLA-C [30], it is the cumulative expression of these genes that was assessed. In porcine and human resting macrophages, SLA-1/2/3/HLA-A/B/C, respectively, and SLA-DRA/HLA-DRA, respectively, are expressed at the same level, which is two to three times the expression level of TNFα (*p* = 0.003 and *p* = 10^− 7^, respectively) (Additional file 4: Table S2; Fig. 5). When the macrophages are activated by LPS/IFNγ, we observed a highly significant over-expression of TNFα both in pigs (*p* = 0.0001) and in humans (*p* = 4.3 10^− 6^). However, we observed a large variability in the expression level of TNFα in pigs, while it is not the case in humans. No significant change in expression level was observed for SLA-1/2/3 and SLA-DRA after macrophage activation, whereas a slight but significant increase in expression was observed for HLA-A/B/C (*p* = 0.013) and HLA-DRA (*p* = 0.005) (Fig. 5). It can therefore be observed that when porcine macrophages are activated, the decompaction of the class I-class III region is accompanied by a strong overexpression of TNFα (class III), whereas in human macrophages, the overexpression of TNFα does not induce the same effect on this distance. In these species, it is the class I-class II region that decompacts in connection with an overexpression of HLA-A/B/C and HLA-DRA. These results provide new elements to reinforce the hypothesis of a different 3D organization and behavior of the MHC in pigs and in humans.

### Comparative analysis of the conformation state of the MHC alleles in each nucleus

**Fig. 6:**
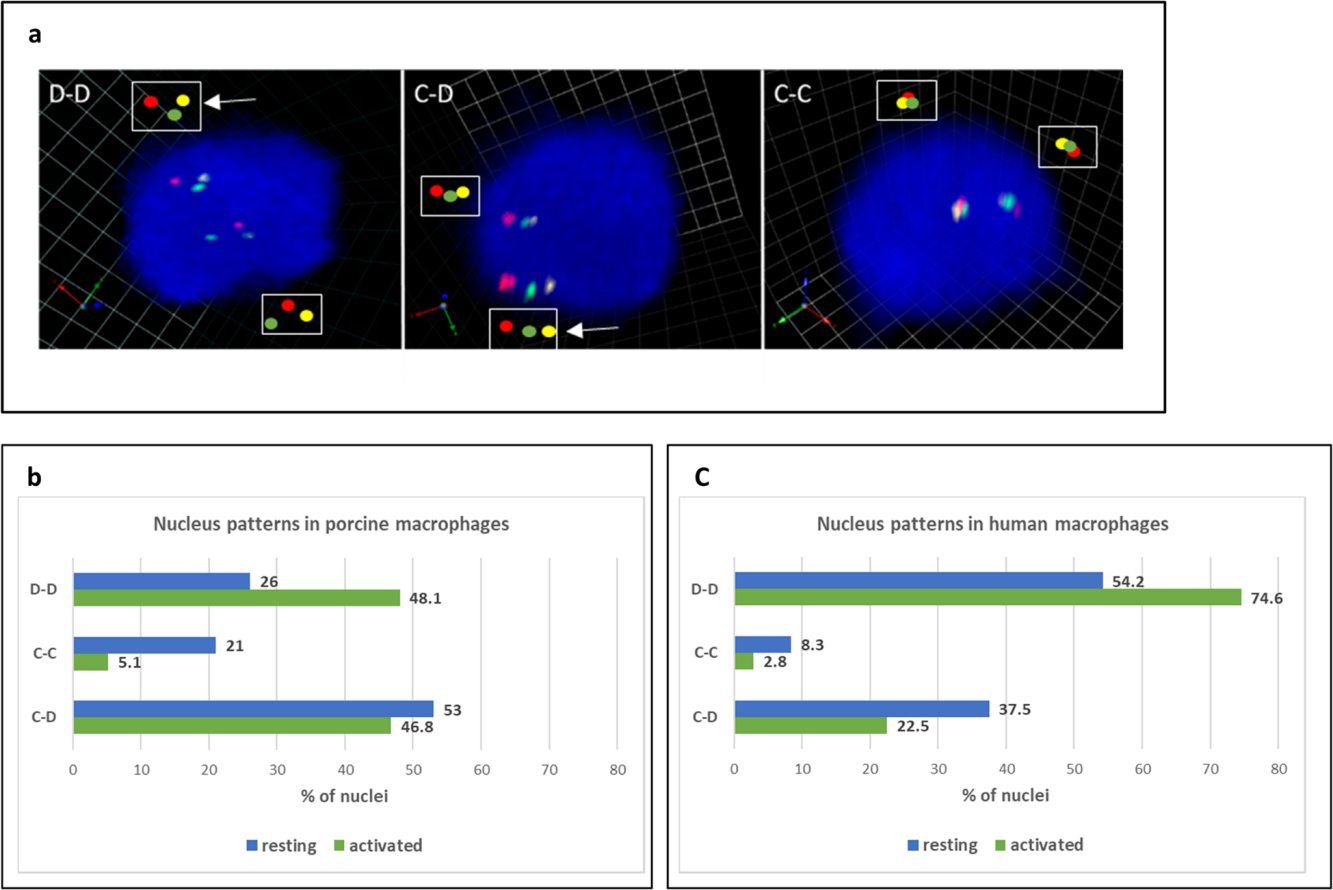
Single nucleus analysis of MHC allele conformations by 3D-FISH; each allele is defined by three fluorescent spots from BAC containing class I labeled in green with Alexa 488; BAC containing class II in red with Alexa 568; and BAC containing class III in yellow with Alexa 633. **a** 3D images displaying examples of the three types of nucleus patterns were shown from the captured images obtained with VOLOCITY software. **b-c** Analysis of LPS/IFNγ activation effects on the allele conformations. Bar graphs represent the percentage of cells in each pattern

We compared the conformation of the two alleles of the same nucleus by performing a single nucleus analysis. Only nuclei in which the two alleles can be clearly differentiated were taken into account. Each allele is defined by three FISH signals for MHC class I, II and III-specific regions, which appear as a compact chromatin cluster (C) or a decompacted chromatin region (D). We first aimed to analyze if the two alleles in each nucleus had the same chromatin compaction state, and then, if the activation of the macrophages induced 3D conformational changes. To address this question, we defined three nucleus patterns using the 3D measurements between the MHC probes (see Methods; Additional file 5: Fig. S3): (i) C-C pattern in which both alleles have a compact cluster conformation; (ii) D-D pattern in which both alleles have a decompacted conformation; and (iii) C-D pattern that represents a mix of the C-C and D-D patterns. Figure 6a illustrates these three patterns. First, to determine whether the two alleles per nucleus have the same conformation in the resting state, we compared the percentage of nuclei harboring the C-D pattern to the one with C-C and D-D patterns under the hypothesis that an allele is as likely to be in the compacted state as in the decompacted one. Consequently, the theoretical value for two alleles of a nucleus to be in the C-D conformation is 50%, while it is 25% for a C-C or D-D conformation. Consequently, the theoretical value for the two alleles to both be in a different (C-D) or similar (C-C or/and D-D) conformation is 50%. In porcine macrophages, we did not observe a difference with an equivalent percentage of nuclei in which the two alleles of the same nucleus have a different conformation (53% of C-D nuclei) and those in which they have the same conformation (47% of (C-C and D-D) nuclei) (Chi squared test for given probabilities, *p*-value = 0.59). Similarly, we observed no significant difference in human macrophages since the two alleles of the same nucleus harbor a different conformation in 37.5% of the nucleus (C-D pattern) and the same conformation in 62.5% of the nucleus (C-C and D-D pattern) (*p*-value = 0.08). It should be noted that when they harbor the same conformation, it is generally a decompacted one. To analyze the effect of LPS/IFNγ activation on these conformations, we then compared the number of nuclei harboring a C-C, D-D or C-D pattern in resting and activated states. The results demonstrate that when porcine macrophages are activated, the number of nuclei with a condensed pattern (C-C) decreases, while the number of nuclei with a D-D pattern significantly increases (*p*-value = 0.001) (Additional file 6: Table S3; Fig. 6b). The same tendency is observed in human cells (*p*-value = 0.048) (Additional file 6: Table S3; Fig. 6c). These results show an increase in decompaction of the MHC region and are consistent with our measurements of the 3D distances between the selected genes.

### Modeling the most probable 3D conformation of the MHC in macrophages and analysis of the impact of cell activation

**Fig. 7:**
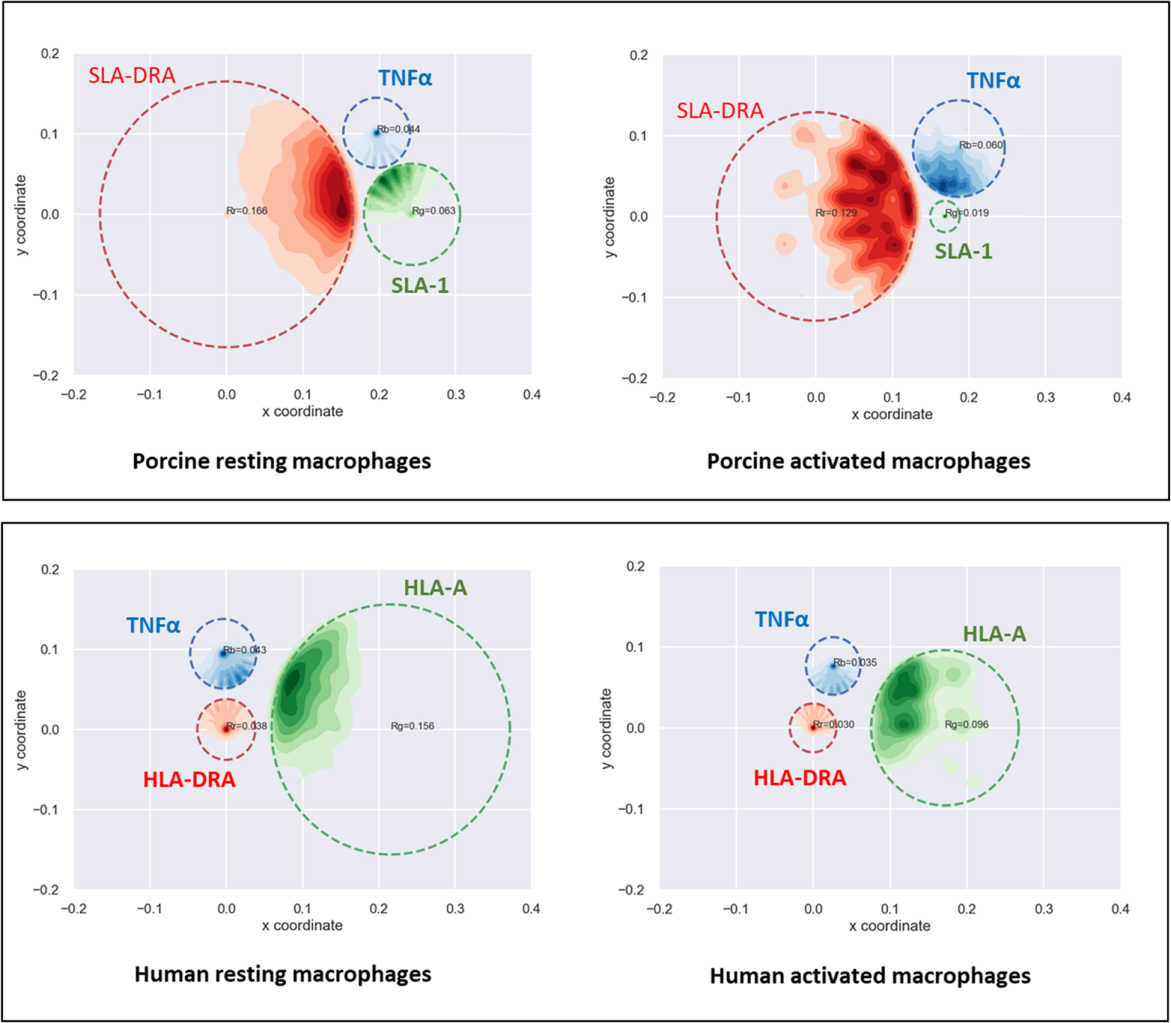
Modeling the relative positions or survival zones (SZs) in resting and activated macrophages of three loci of the MHC whose positions are spatially linked. The loci are SLA-1/HLA-A (class I) in green, TNFα (class III) in blue, and SLA-DRA/HLA-DRA in red. The mathematical approach makes it possible to determine the specific area (SZ) in which each locus moves in resting and activated macrophage nuclei. These SZ are represented by colored dotted circles. The radii (R_g_, R_b_ and R_r_) of the SZs are indicated for each locus. All the different locus positions (X,Y) are reconstructed inside survival zones. The Python module, Seaborn, is used for 2D kernel density estimation of reconstructed points (https://seaborn.pydata.org/index.html)

To support our experimental observations and take the organization of the complex as a whole into account by extending the analysis of the 3D distances two-by-two, we applied an optimized version of the three-loci algorithm [31]. The goal of this method is to determine the MHC conformation from the relative positions of three loci measured in pig and human macrophages following 3D FISH experiments*.* In each nucleus, we observed distinct states of the chromatin fiber, and using the mathematical algorithm, we can assess a physical constraint between three loci within the same chromosome segment. The mathematical model is based on the assumption that each labeled locus moves in a specific area called a survival zone (SZ). Thus, we can determine the survival zones of each gene SZ_SLA-1(HLA-A),_ SZ_TNFα_ and SZ_SLA-DRA (HLA-DRA)_ within a single cell in pigs and humans, respectively (Additional file 7: Fig. S4; Fig. 7). While class III SZ (SZ TNFα) is similar in resting porcine and human cells (*R* = 0.44 and *R* = 0.43, respectively), the SZ of the two other classes (SZ_SLA-1(HLA-A)_ and SZ_SLA-DRA (HLA-DRA_) show notable differences between pigs and humans. Indeed, the class II region is markedly more constrained in humans than in pigs, whereas the opposite is observed for class I, which is more constrained in pigs. All this reinforces the hypothesis of a different 3D organization of SLA and HLA complexes in resting cells. When the macrophages are activated, there is an overall decrease in the space occupied by the MHC in both species. However, some differences are noteworthy. In porcine cells, the SZ_SLA-DRA_ is reduced from *R* = 0.166 to *R* = 0.129, but the most striking feature concerns SLA-1. The size of its SZ is considerably reduced (from *R* = 0.063 to *R* = 0.019) and its position changes substantially with a tendency to move towards SLA-DRA (Fig. 7). Globally, these observations reflect that the freedom of motion of the loci is reduced upon activation. The reduction of the SZs is consistent with the finding that the area explored by actively transcribed loci is confined upon transcription activation [32]*.* In human macrophages, we observed little change when the cells are activated except for the SZ of HLA-A, which is reduced from *R* = 0.156 to *R* = 0.096. Since the MHC is located on the same chromosomal arm, gene activation may require less effort to fold this domain upon LPS/IFNγ activation.

## Discussion

In this study, we analyzed the 3D conformation of MHC in macrophages, one of the most gene-dense genomic regions in mammals. Due to its key function in immunity, this complex has been widely investigated in terms of sequence, genetic diversity, gene function and expression, both in humans [6] and in pigs [8]. However, few studies have reported on its 3D organization in the nucleus of immune cells like macrophages, even though the important role of this organization on the regulation of gene expression has been widely demonstrated in many cell types [13, 33].

### Intermediate radial positioning of a gene-dense region

To investigate how the consecutive genes that compose the MHC are spatially arranged in interphase nuclei, we selected BACs containing genes representative of each MHC region in human and in pig macrophages to first analyze their radial position and then their gene-to-gene distances. We found that MHC class I, class II and class III regions had a non-random spatial organization in both species (Fig. 2). We showed that these three genes are at the same radial position in porcine macrophages, probably located in an intermediate position rather than inside or on the periphery of the nucleus. The same situation is observed in human macrophages for genes in class II and class III with an intermediate radial position, whereas the class I gene (HLA-A) harbors a significantly different position, with a clear tendency to be more toward the periphery. Considering that these genes are located in a gene-dense genomic region and are expressed in resting macrophages, a more central position in the nucleus would have been expected. Indeed, a neighborhood effect has been suggested by the observation that radial positioning often correlates with local gene density, with locally gene-dense regions preferentially having an internal position. This was first shown at the whole chromosome level with gene-poor chromosomes positioned toward the nuclear periphery and gene-rich chromosomes inside the nucleus [34], and then at the single gene level [35]. In addition, several studies have suggested that a functional link exists between gene activity and radial positioning, where active genes are preferentially located inside the nucleus (reviewed in [36]). However, this is not a universal hallmark. While some authors have demonstrated that locations closer to the nuclear periphery are not incompatible with active transcription [37, 38], others have even suggested that there is not necessarily a correlation between gene activity level and radial positioning [39]. We also previously demonstrated in porcine macrophages that there is not always a link between radial positioning and variations of expression since down-regulated genes can occupy the same radial position as up-regulated genes [28]. The data presented in this study are consistent with this hypothesis considering the radial positioning of the three MHC genes analyzed and their level of expression in resting macrophages in both species.

### Different 3D organization of the MHC in humans and pigs

The 3D gene-to-gene distance measurements allowed us to demonstrate that the MHC class I, class II and class III genes have a non-linear organization in the nuclei of macrophages. Indeed, in porcine macrophages, our results indicated that SLA-DRA tends to be at an equivalent distance of SLA-1 and TNFα (Fig. 3a; Additional file 2: Table S1) in contrast to the genomic map where it is at a physical distance of 1.9 Mb and 1.1 Mb from SLA-1 and TNFα, respectively (Fig. 1). On the contrary, in human macrophages, there is a good correlation between the distances in megabases and the 3D measured distances (Fig. 1; Additional file 2: Table S1). The mean values of the internal angles were calculated using all 3D distance measurements. This allowed us to propose a different 3D conformation for the MHC in porcine and human macrophages (Fig. 3a).

We then investigated whether the activation process modifies these conformations. Our results demonstrate that upon LPS/IFNγ activation, a decompaction of the chromatin is observed in the HLA and SLA complexes but it does not affect the same regions. In fact, it is the class I (HLA-A)-class II (HLA-DRA) distance that significantly increases in human activated macrophages, whereas it is the class I (SLA-1)–class III (TNFα) distance in the porcine cells (Fig. 4a-b). The differences we observed both in the 3D conformation of the human and porcine MHC and in the effects following macrophage activation are not surprising when all the elements of comparison between the human and porcine MHC are accounted for. Considering human and swine MHC, there is a high overall level of conserved synteny. These two genomic regions are delimited by MOG upstream from the class I region and RING1 downstream from the class II region [8]. The anchor genes that delimit the three regions are found in both species: (i) MOG and MCCD1 for class I; (ii) MCCD1 and BTNL6 for class III; and (iii) BTLN2 and RING1 for class II. However, there are several points in which SLA and HLA complexes significantly differ [40]. The first one concerns the size and organization of the class I region. It is organized into two clusters and spans 1.06 Mb in swine, whereas it is organized into three clusters and spans 1.89 Mb in humans. This difference in size corresponds to a segment of 300 kb present in the HLA and absent in the SLA complex [10]. The second important difference has to do with the position of the centromere. The porcine MHC is unique in that the class II region is separated from the class III and class I regions by the centromere. This could imply major differences in the 3D organization of the human and porcine MHC. The importance of centromeres in the genome 3D organization has only just begun to be recognized [41]. It has, at least in part, probably to do with the nature of their sequences. Centromeres are associated with large arrays of tandemly alpha satellite DNA sequences flanked on both sides by compact blocks of constitutive heterochromatin. An example of their strong topological impact has been reported through Hi-C data that have demonstrated that centromeres could act as a barrier to intrachromosomal arm interactions [41]. The pericentromeric heterochromatin would also have a preventive role, hindering the invasion of the centromeric region in euchromatin [42]. The conformational changes we observed in activated compared to resting porcine macrophages by applying the three-loci algorithm could indicate that chromatin reorganization within the porcine MHC is greater due to the physical barrier caused by the centromere.

### Conformation of MHC alleles in the same nucleus

It has been shown that because genome organization is inherently flexible, the two alleles in a cell may be differentially organized at the chromatin architecture level. The most striking examples are often associated with cellular processes that require persistent differences in allele function, such as dosage compensation, imprinted genes, and monoallelically expressed genes [21]. However, using high-throughput methods (genome-wide Hi-C and large imaging datasets) to study genomic interactions in the nucleus, the authors have extended these specific examples and have highlighted the high level of heterogeneity in spatial genome organization among individual cells as well as between alleles in the same cell [21]. Using the 3D distance measurements between the MHC target genes, we defined a conformation state for each allele. Even if we do not have allele-specific probes, it was possible to compare the conformation of the two alleles of the same nucleus by performing a single nucleus analysis. This also allowed us to pinpoint a heterogeneity in chromatin conformation between alleles in the same nucleus, both in porcine and human resting macrophages, with as many nuclei in which the two alleles have a different conformation as nuclei in which they have the same conformation. This variability in allele conformation could be linked to their expression status. Indeed, it has been demonstrated, by investigating single-cell transcriptome, that active transcription generally occurs in bursts ranging from minutes to hours in length. Given its inherent stochastic nature, transcriptional bursting is not coordinated between homologous alleles. Thus, at any given point in time, only one allele may be actively transcribing for a given gene [43–45]. This pattern of punctuated gene transcription has been observed even for genes with very high levels of expression. The chromatin in the vicinity of actively expressed genes are less condensed and in a more open state than in the vicinity of non-expressed genes. This is to facilitate the access of the transcription machinery to chromatin.

When macrophages are activated by LPS/IFNγ, we observe a very significant increase in the number of alleles, showing a decompacted conformation both in humans and in pigs. This is associated with the up-regulation of the three MHC target genes in human macrophages and with the up-regulation of TNFα in porcine cells. These results are in agreement with those we previously obtained. We in fact demonstrated that when macrophages are LPS/IFNγ activated, several cytokines, including TNFα, have a tendency to be outside their chromosome territories [28], suggesting that the transcriptional activation leads to decompaction or the disturbance of chromatin loops to facilitate transcription. Several studies focusing on the MHC have also already demonstrated such a phenomenon after up-regulation by IFNγ in cell types other than immune cells, including fibroblasts and fibrosarcoma-type cancer cells [24, 46, 47]. They reported that chromatin carrying the entire MHC undergoes massive higher-order remodeling mediated by the transcription factor STAT1. However, they made the assumption that external loops might not be an absolute requirement for transcription per se since even within a population of cells that is expressing the locus at a very high level, the MHC was found on an external loop in a maximum of 35% of the chromosome territories [46]. Our study suggests that in immune cells, this phenomenon of MHC chromatin remodeling upon LPS/IFNγ activation is probably more widespread since we observed a high percentage of MHC alleles in a decondensation state both in human and in porcine macrophages. This phenomenon is probably related to their function in the immune response since they are the first actors in the inflammatory process.

In further research, it would be interesting to extend this single-cell approach that revealed a higher percentage of decondensed MHC alleles in activated macrophages by performing DNAse1 hypersensibility assay which allows targeting the regions of chromatin that have lost their compact structure. This kind of approach has been performed to map the DNAse 1 hypersensitive regions in the MHC of Primates to determine the disease-causing variants for most major histocompatibility complex (MHC)-associated diseases [48]. Similarly, as we detected a strong overexpression of TNFα in human and porcine macrophages following LPS/IFNγ activation, a comparative study of its epigenetic profile [49] (both at the DNA methylation and histone marks levels) in resting and activated cells would further our results.

## Conclusion

This study adds new data on the 3D organization in macrophage nuclei of one of the most important regions of the genome since its contains the MHC, which plays a crucial role in the immune response. We showed that this complex adopts a different 3D conformation in porcine and in human macrophages. The activation of the cells by LPS/IFNγ modifies this organization more noticeably in porcine cells. The presence of the centromere within the SLA complex, which is a particularity in pigs, could be a factor that could explain, at least in part, the observed differences.

## Methods

### Ethics statement

Our experiment was conducted in accordance with the French national regulations for human care and use of animals in research. Swine blood samples were collected on Large White pigs from UMR INRAE Toxalim under the experimentation agreement number TOXCOM/0020/PP AL. Human blood samples from healthy donors were provided by Etablissement Français du sang (Toulouse, France).

### Cell preparation and activation

Porcine monocytes were isolated as previously described [28] with few modifications. Briefly, peripheral blood mononuclear cells (PBMCs) were separated by density gradient centrifugation (1.077 g/l, Lymphoprep, Eurobio,les Ulis, France) for 30 min at 800 g at room temperature. PBMCs were collected and submitted to a lysis solution (NH_4_Cl 0.15 M KHCO_3_ 0.01 M–EDTA Na_2_ 1 μM) for 5 min to eliminate red cells. One- × 10^8^ cells were distributed on CellBIND® flask in RPMI-1640 supplemented with penicillin-streptomycin (100 U/ml) and without FCS to allow for their adherence. After 4 h at 37 °C in humidified atmosphere 5% CO_2_, medium was replaced by RPMI-1640 supplemented with 10% FCS, 1% non-essential amino acids (Sigma-Aldrich, Saint Quentin Fallavier, France) and 10% of CSF-1 growth factor (LADMAC cell medium, [50]) for the generation of monocyte-derived macrophages. Flasks were placed at 37 °C 5% CO_2_ for 4 days. The cells were then submitted to labelling with antihuman CD14 antibody (MACS CD14 microbeads, Miltenyi Biotec, Paris, France). The CD14+ cells were selected with an AutoMACS separation column (Miltenyi Biotec) according to the manufacturer’s instructions. Macrophages were suspended in RPMI-1640 to a final concentration of 5.10^6^ cells/ml.

Human CD14+ monocytes were isolated from blood as previously described [51]. They were then distributed on glass coverslips at 1–1.5 × 10^6^ cells in 6-well plates in RPMI 1640 without FCS to allow for their adherence. After 2 h at 37 °C in humidified 5% CO_2_ atmosphere, the medium was replaced by RPMI 1640 containing 10% heat-inactivated FCS, antibiotics and 20 ng/ml M-CSF (PeproTech, Rock Hill, NJ, USA). They were maintained in culture for 6–8 days to differentiate into macrophages.

Human and porcine macrophage samples were divided in two: one part was incubated in culture medium (resting batch), the other in a culture medium supplemented with LPS (10 μg/ml) and IFNγ (1 ng/ml, activated batch) for 3 h at 37 °C according to the activation conditions defined previously [28].

### 3D-FISH experiments

#### Slides for 3D-FISH

A suspension of 5 × 10^6^ cells/ml of porcine macrophages in RPMI 1640 was applied to poly-L-lysine slides for 10 min for cell adhesion. 3D fixation was performed at room temperature for 10 min in 4% paraformaldehyde freshly made followed by a short wash in PBS. Slides were then immersed in 20% glycerol solution for 30 min and stored at − 80 °C.

Glass-coverslips with spread human macrophages were placed 10 min in 4% paraformaldehyde followed by a short wash in PBS and used immediately for 3D FISH without freezing.

#### DNA probes

The porcine BAC clones containing MHC class I (SLA-1, SBAB-490B10) [52], class II (SLA-DRA, SBAB-591C4) and class III (TNFα, SBAB-493A6) genes [10] and the one labelling the SSC7 centromere (SBAB-437A9) were isolated from a swine BAC library [53] (CRB-Anim, INRA, 2018. Biological Resource Centres for domestic animals of AgroBRC, doi: 10.15454/1.5613785622827378E12). The human BAC clones containing orthologous genes were purchased from BACPAC Resources at Children’s Hospital and Research Center [54] (RPCI human BAC library 11, Oakland, USA: https://bacpacresources.org;): MHC class I (HLA-A, RP11-192H11), class II (HLA-DRA, RP11-379F19), class III (TNFα, RP11-184F16). The presence of the selected genes in each BAC clone was verified by PCR using the primers listed in Table 1. Approximatively 50 ng of BAC DNAs were random priming labelled using the Bioprime DNA labeling kit (Invitrogen, Cergy-Pontoise, France). For multiple-label experiments, BAC containing class I and BAC containing class II were directly labelled by incorporation of dUTP Alexa 488 and dUTP Alexa 568 (Invitrogen) respectively. The BAC containing TNFα (class III region) and the BAC specific for SSC7 centromere were labelled with biotinylated dUTP (Roche) revealed with streptavidine-Alexa-633 (Invitrogen). The same procedure is applied to porcine and human BAC probes. Products from the labelling reaction of the three BACs in each species were pooled and precipitated with porcine or human Cot-1 DNA (Applied Genetics Laboratories, Melbourne, USA) and salmon sperm DNA (Eurobio, Les Ulis, France). Probes were dropped onto macrophage slides or coverslips at a final concentration of 100 ng/μl in a hybridization buffer. The specificity of the probes was previously tested by 2D-FISH on porcine or human metaphase spreads (Additional file 1: Fig. S1) prepared from lymphocytes according to protocols, which had previously been described [55].

#### 3D FISH experiment

3D FISH experiments were carried out as previously described [28] both for porcine and human macrophages. Briefly, slides were successively immersed in 0.5% Triton X-100-Saponin solution and Tris-HCl 0.1 M pH 7.2. Cells were then freeze-thawed six times in liquid nitrogen, treated with 200 μg/ml RNase and 0.1 N HCl solution. Finally, slides were incubated in 2XSSC-50% formamide for at least 3 days at 4 °C. Cells and probes were simultaneous denatured 5 min at 72 °C and incubated 72 h at 37 °C in a DAKO hybridizer. Post-hybridization washes were performed twice in 2X SSC at room temperature for 15 min, then three times for 15 min in 2X SSC, 50% formamide pH 7.0 at 45 °C and finally, three times for 15 min in 0.1X SSC at 45 °C. Nuclei were counterstained with 4’,6’diamidino-2-phenylindole in Vectashield medium (Vector Laboratories, Burlingame, USA).

### Confocal microscopy and image analyses

Confocal microscopy was carried out using a Leica TCS SP2 confocal microscope (Leica Instruments, Heidelberg Germany) equipped with an oil immersion objective (plan achromatic 63× N.A. = 1.4). The Z-stacks were acquired at 1024 × 1024 pixels per frame using an 8-bit pixel depth for each channel at a constant voxel size of 0.079 × 0.079 × 0.244 μm. Typically, a stack of 45 confocal planes was acquired. Segmentations and 3D measurements between objects were done using NEMO software [29]. The radial position of the genes was calculated using the distance between the gene center and the nucleus center normalized by the nucleus local radius. The 3D gene center-to-gene center distances were normalized by the nucleus local diameter for each scored cell. To compare the conformation of the two alleles in each nucleus, we defined three nucleus patterns: (i) D-D pattern (Decompacted) when the three loci that define each allele were found separed by a distance d_1_ ≥ 2r, given that r is the radius of the fluorescent spot that represents the loci, (ii) C-C pattern (Compacted) when the three loci that define each allele were found separed by a distance d_2_ ≤ 2r and (iii) C-D pattern when the three loci that define one allele were found separed by a distance d_2_ ≤ 2r (C) while the distances between the three loci that define the other allele were d_1_ ≥ 2r (D) (Additional file 5: Fig. S3).

### Quantitative real time RT-PCR

The primers for quantitative real time PCR are those used for the control of the BAC specificity as they have been chosen in exons (Table 1). The β-2-Microglobulin was chosen as the internal reference gene. Resting and activated macrophage samples of 5 pig and 5 human donors were used. Total RNA was isolated with NucleoSpin® RNA kit (Macherey Nagel) according to the manufacturer instruction. The integrity and quantification of the RNA samples were assessed using an Agilent Bioanalyzer 2100 with a RNA 6000 Nano Lab. RNA (0.5 μg) with a RIN score between 8 and 10 were reverse transcribed using random primers and Superscript III enzyme (Life Technologies). The resulting cDNA samples were diluted 1/25. QPCR was performed in duplicate with 2 μl of the dilution in a final volume of 8 μl using SYBR™ Green PCR Master Mix (Applied biosystems) on a QuantStudio6 real-time PCR System (Thermo Fisher Scientific) as previously described [56]. The efficiency of real-time PCR amplification was calculated for each primer pair using six serial dilution points from a cDNA pool including pure resting and activated cDNA samples from resting and activated samples (1:5; 1:10; 1:20; 1:40; 1:80; 1: 160). After determination of the threshold cycle (Ct) for each sample, the PFAFFL method was applied to calculate the relative expression of each gene [57] using the cDNA pool as calibrator sample.

### 3D modeling of the MHC conformation: inferring survival zone radius

In our model, we assumed that in the time interval *Δt*, the genes move into regions whose confinement radii are *R*_*r*_*, R*_*g*_ and *R*_*b*_ (Additional file 7: Fig. S4). These regions are called locus survival zones. Indices *r, g* and *b* correspond to the loci: *r = SLA-DRA, g = SLA-1*, *b = TNFα* for the pig and *r = HLA-DRA, g = HLA-A, b = TNFα* for the human.

If we denote by ((*R* − *G*)_*i*_)_*i* = 1. . *n*_, ((*G* − *B*)_*i*_)_*i* = 1. . *n*_ and ((*R* − *B*)_*i*_)_*i* = 1. . *n*_ different distances between the loci, we can set.

$$ {\displaystyle \begin{array}{c}{d}_{rgmin}=\mathit{\min}\left({\left({\left(R-G\right)}_i\right)}_{i=1..n}\right)\ {d}_{rgmax}=\mathit{\max}\left({\left({\left(R-G\right)}_i\right)}_{i=1..n}\right)\\ {}{d}_{rbmin}=\mathit{\min}\left({\left({\left(R-B\right)}_i\right)}_{i=1..n}\right)\ {d}_{rbmax}=\mathit{\max}\left({\left({\left(R-B\right)}_i\right)}_{i=1..n}\right)\\ {}{d}_{gbmin}=\mathit{\min}\left({\left({\left(G-B\right)}_i\right)}_{i=1..n}\right){d}_{gbmax}=\mathit{\max}\left({\left({\left(G-B\right)}_i\right)}_{i=1..n}\right)\end{array}} $$

Where *n* is the number of cells, **min()** is the minimum function and **max()** is the maximum function.

Survival zones radii can be expressed by the expressions:

$$ {\displaystyle \begin{array}{c}{R}_g=\frac{d_{rgmax}+{d}_{gbmax}+{d}_{rbmin}-{d}_{rbmax}-{d}_{rgmin}-{d}_{gbmin}}{4}\\ {}{R}_b=\frac{d_{rbmax}+{d}_{gbmax}+{d}_{rgmin}-{d}_{rgmax}-{d}_{rbmin}-{d}_{gbmin}}{4}\\ {}{R}_r=\frac{d_{rgmax}+{d}_{rbmax}+{d}_{gbmin}-{d}_{gbmax}-{d}_{rgmin}-{d}_{rbmin}}{4}\end{array}} $$

## Supplementary Information

**Additional file 1 Fig. S1.** BAC probe specificity control by 2D-FISH on porcine metaphases. **(a)** class I (SLA-1, SBAB-490B10) in green; class II (SLA-DRA, SBAB-591C4) in red; and class III (TNFα, SBAB-493A6) in yellow. **(b)** porcine chromosome 7 centromere in red (SBAB-437A9). **(c)** BAC probe specificity control by 2D-FISH on human metaphases: class I (HLA-A, RP11-192H11) in green; class II (HLA-DRA, RP11-379F19) in red; and class III (TNFα, RP11-184F16) in yellow.

**Additional file 2 Table S1.** Mean values of 3D normalized center-to-center distances and angles calculated using center-to-center distances.

**Additional file 3 Fig. S2.** 3D BAC center-to-center distances in resting and activated swine macrophages. Cumulative frequency graphs of the normalized center-to-center distances between: (i) MHC class III (TNFα) and SSC7 centromere in red; (ii) MHC class II (SLA-DRA) and SSC7 centromere in green. Pairwise comparisons (*p*-values) of cumulative 3D distance distributions in resting and activated macrophages (Student’s t-test) are indicated in each graph.

**Additional file 4 Table S2.** Comparison of gene expression level in resting macrophages. mRNA expression level, analyzed by RT-qPCR, were pairwise compared with one-way ANOVA model with repetitions.

**Additional file 5 Fig. S3.** Schematic representation of the method used to define the conformation of each allele: given that r1 and r2 are the radii of two fluorescent spots, when the 3D distance between two spot centers (d1) is greater than r1 + r2, the allele is assumed to be decompacted (D), whereas when the 3D distance (d2) between two spot centers is less than r1 + r2, the allele is assumed to be compacted (C).

**Additional file 6 Table S3.** Nucleus-by-nucleus analysis of pairs of allele conformations and of the effects of LPS/IFNγ activation.

**Additional file 7 Fig. S4.** Schematic representation of genes positioned inside survival zones (SZ) as determined by the application of the three-loci algorithm. The probable positioning of three genes (blue, red and green dots) is represented in two cells as an example (in cell 1 shown by dotted lines, and in cell 2 by continuous lines). The different positions of each gene make it possible to define its SZ represented by a circle of the same color. The radius of the circle defined for each locus is denoted by Rb for the blue dot, Rr for the red dot and Rg for the green dot.

## Data Availability

The datasets supporting the conclusions of this article are included within the article, its additional files. All relevant raw data have been deposited at https://data.inrae.fr/privateurl.xhtml?token=abaff29a-cdcd-4625-b51c-51dfa69f35ff

## Abbreviations

2D FISH: Two-dimensional fluorescence in situ hybridization
3D FISH: Three-dimensional fluorescence in situ hybridization
BAC: Bacterial Artificial Chromosome
CT: Chromosome Territory
DAPI: 4’, 6’ diamidino-2-phenylindole
SSC: *Sus scrofa* domestica
MHC: Major Histocompatibility Complex
MOG: Myelin Oligodendrocyte Glycoprotein
RING1: Ring finger protein 1
MCCD1: Mitochondrial Coiled-Coil Domain 1
BTNL2: Butyrophilin-like 2
BTNL6: Butyrophilin-like 6
