# Supplementary figures and images for: The 3D nuclear conformation of the major histocompatibility complex changes upon cell activation both in porcine and human macrophages
Source: BMC Mol Cell Biol. 2021 Sep 14;22:45. doi: 10.1186/s12860-021-00384-4 (PMC8442435; doi:10.1186/s12860-021-00384-4)

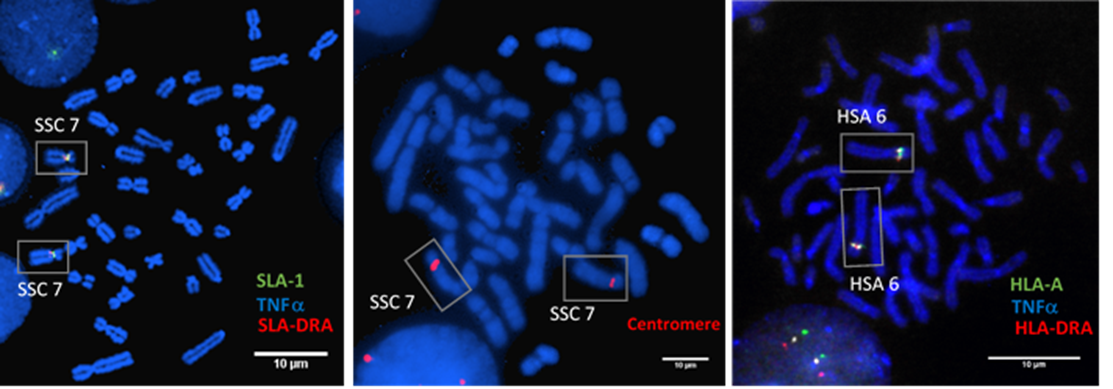


a b c

Additional file 1: Figure S1

Supplement: Supplementary file 1 — Additional file 1 Fig. S1. BAC probe specificity control by 2D-FISH on porcine metaphases. (a) class I (SLA-1, SBAB-490B10) in green; class II (SLA-DRA, SBAB-591C4) in red; and class III (TNFα, SBAB-493A6) in yellow. (b) porcine chromosome 7 centromere in red (SBAB-437A9). (c) BAC probe specificity control by 2D-FISH on human metaphases: class I (HLA-A, RP11-192H11) in green; class II (HLA-DRA, RP11-379F19) in red; and class III (TNFα, RP11-184F16) in yellow. [file 12860_2021_384_MOESM1_ESM.docx]

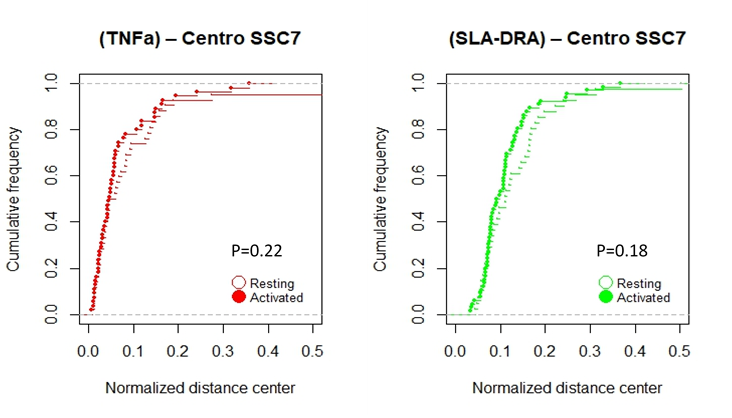


Additional file 3: Figure S2

Supplement: Supplementary file 3 — Additional file 3 Fig. S2. 3D BAC center-to-center distances in resting and activated swine macrophages. Cumulative frequency graphs of the normalized center-to-center distances between: (i) MHC class III (TNFα) and SSC7 centromere in red; (ii) MHC class II (SLA-DRA) and SSC7 centromere in green. Pairwise comparisons (p-values) of cumulative 3D distance distributions in resting and activated macrophages (Student’s t-test) are indicated in each graph. [file 12860_2021_384_MOESM3_ESM.docx]

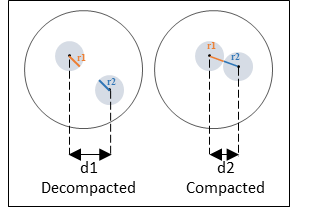


Additional file 5: Figure S3

Supplement: Supplementary file 5 — Additional file 5 Fig. S3. Schematic representation of the method used to define the conformation of each allele: given that r1 and r2 are the radii of two fluorescent spots, when the 3D distance between two spot centers (d1) is greater than r1 + r2, the allele is assumed to be decompacted (D), whereas when the 3D distance (d2) between two spot centers is less than r1 + r2, the allele is assumed to be compacted (C). [file 12860_2021_384_MOESM5_ESM.docx]

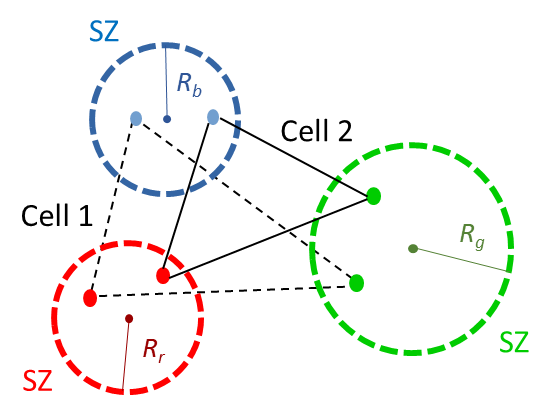


Additional file 7: Figure S4

Supplement: Supplementary file 7 — Additional file 7 Fig. S4. Schematic representation of genes positioned inside survival zones (SZ) as determined by the application of the three-loci algorithm. The probable positioning of three genes (blue, red and green dots) is represented in two cells as an example (in cell 1 shown by dotted lines, and in cell 2 by continuous lines). The different positions of each gene make it possible to define its SZ represented by a circle of the same color. The radius of the circle defined for each locus is denoted by Rb for the blue dot, Rr for the red dot and Rg for the green dot. [file 12860_2021_384_MOESM7_ESM.docx]
