# Supplementary material for: The 3D nuclear conformation of the major histocompatibility complex changes upon cell activation both in porcine and human macrophages
Source: BMC Mol Cell Biol. 2021 Sep 14;22:45. doi: 10.1186/s12860-021-00384-4 (PMC8442435; doi:10.1186/s12860-021-00384-4)
Supplement: Supplementary file 2 — Additional file 2 Table S1. Mean values of 3D normalized center-to-center distances and angles calculated using center-to-center distances. [file 12860_2021_384_MOESM2_ESM.docx]

|  | | **Mean values of normalized distances**  (standard deviation) | | | **Mean values of angles**  (degree) | | |
| --- | --- | --- | --- | --- | --- | --- | --- |
| **Species** | **Macrophages** | **Class I-II (a)** | **Class I-III (c)** | **Class II-III (b)** | **A**  **(class I-II)** | **B**  **(class II-III)** | **C**  **(class I-III)** |
| **Pig** | **resting** | 0.118  (0.07) | 0.052  (0.03) | 0.117  (0.06) | 76 | 77 | 27 |
|  | **activated** | 0.120  (0.06) | 0.06  (0.03) | 0.120  (0.06) | 77 | 76 | 28 |
|  |  |  |  |  |  |  |  |
| **Human** | **resting** | 0.129  (0.06) | 0.117  (0.06) | 0.064  (0.03) | 93 | 31 | 56 |
|  | **activated** | 0.144  (0.05) | 0.117  (0.05) | 0.067  (0.03) | 101 | 27 | 52 |

Additional file 2: Table S1
