# Supplementary material for: The 3D nuclear conformation of the major histocompatibility complex changes upon cell activation both in porcine and human macrophages
Source: BMC Mol Cell Biol. 2021 Sep 14;22:45. doi: 10.1186/s12860-021-00384-4 (PMC8442435; doi:10.1186/s12860-021-00384-4)
Supplement: Supplementary file 4 — Additional file 4 Table S2. Comparison of gene expression level in resting macrophages. mRNA expression level, analyzed by RT-qPCR, were pairwise compared with one-way ANOVA model with repetitions. [file 12860_2021_384_MOESM4_ESM.docx]

|  | PIG | HUMAN |
| --- | --- | --- |
| Comparison of gene expression level in resting macrophages | *p-value* | *p-value* |
| Class II (DRA) – Class I (SLA-1/2/3-HLA-A/B/C) | 0.86 | 0.218 |
| Class III (TNFα) - Class I (SLA-1/2/3-HLA-A/B/C) | 0.0033 | 10^-7^ |
| Class III (TNFα) - Class II (DRA) | 0.0082 | 10^-7^ |

Additional file 4: Table S2
