# Supplementary material for: The 3D nuclear conformation of the major histocompatibility complex changes upon cell activation both in porcine and human macrophages
Source: BMC Mol Cell Biol. 2021 Sep 14;22:45. doi: 10.1186/s12860-021-00384-4 (PMC8442435; doi:10.1186/s12860-021-00384-4)
Supplement: Supplementary file 6 — Additional file 6 Table S3. Nucleus-by-nucleus analysis of pairs of allele conformations and of the effects of LPS/IFNγ activation. [file 12860_2021_384_MOESM6_ESM.docx]

| **Analysis of LPS-IFNγ activation effect on allele conformations** | | | | | | |
| --- | --- | --- | --- | --- | --- | --- |
| **Species** | **Macrophage state** | **Total number of nuclei analyzed** | **Nucleus pattern**  (Number of nuclei, %) | | | **p-value**  (χ2 test by Monte Carlo) |
|  |  |  | C-D^1^ | C-C^2^ | D-D^3^ |  |
| Pig | resting | 87 | 46 (53%) | 18 (21%) | 23 (26%) | 0.001 |
|  | activated | 79 | 37 (46.8%) | 4 (5.1%) | 38 (48.1%) |  |
|  |  |  |  |  |  |  |
| Human | resting | 48 | 18 (37.5%) | 4 (8.3%) | 26 (54.2%) | 0.048 |
|  | activated | 71 | 16 (22.5%) | 2 (2.8%) | 53 (74.6%) |  |

^1^ C-D = Compacted-Decompacted pattern

^2^ C-C = Compacted- Compacted pattern

^3^ D-D = Decompacted-Decompacted pattern

Additional file 6: Table S3
